# Short-time intensive insulin therapy upregulates 3 beta- and 17 beta-hydroxysteroid dehydrogenase levels in men with newly diagnosed T2DM

**DOI:** 10.3389/fendo.2022.894743

**Published:** 2022-07-19

**Authors:** Yun Hu, Ying Wang, Ting-ting Cai, Lu Liu, Dong-mei Li, Jian-hua Ma, Bo Ding

**Affiliations:** ^1^ Department of Endocrinology, Nanjing First Hospital, Nanjing Medical University, Nanjing, China; ^2^ Department of Endocrinology, Wuxi People’s Hospital Affiliated to Nanjing Medical University, Wuxi, China; ^3^ Department of Endocrinology, Chunjiang People’s Hospital, Changzhou, China

**Keywords:** beta-hydroxysteroid dehydrogenase, diabetes mellitus, dehydroepiandrosterone sulfate, testosterone, insulin

## Abstract

**Objective:**

Our previous study has found that short-term intensive insulin therapy in patients with newly diagnosed type 2 diabetes mellitus (T2DM) increased serum testosterone levels, but the underlying mechanisms remain unclear.

**Design and methods:**

In this self-controlled study, 43 men with newly diagnosed drug naïve T2DM, aged 18-60 years, with HbA_1c >_9.0% were treated with continuous subcutaneous insulin infusion (CSII) to normalize blood glucose within one week. Venous blood specimens were collected for measuring of serum total testosterone, dehydroepiandrosterone sulfate (DHEA-S), 3β- and 17β-hydroxysteroid dehydrogenase (3β- and 17β-HSD) concentrations before and after insulin therapy.

**Results:**

Testosterone increased from 13.0 (11.3, 14.6) nmol/L to 15.7 (13.9, 17.5) nmol/L after intensive insulin therapy (*p*<0.001), while the levels of DHEA-S decreased significantly after treatment (from 6.5 (5.7, 7.3) μmol/L to 6.0 (5.3, 6.7) μmol/L, *p*=0.001). The ratio of testosterone/DHEA-S increased significantly (2.4 (2.0, 2.8) vs. 3.1 (2.6, 3.7) nmol/μmol, *p*<0.001). After blood glucose normalization with the short-term CSII therapy, 3β-HSD increased from 11.0 (9.5, 12.5) pg/mL to 14.6 (13.5, 15.7) pg/mL, *p*=0.001, and 17β-HSD increased from 20.7 (16.3, 25.2) pg/mL to 28.2 (23.8, 32.5) pg/mL, *p*=0.009.

**Conclusions:**

Blood glucose normalization *via* short-term intensive insulin therapy increases plasma total testosterone levels in men with newly diagnosed type 2 diabetes, associated with a decreased level of DHEA-S, probably because of the enhanced conversion from DHEA to testosterone catalyzed by 3β-HSD and 17β-HSD.

## Introduction

Increasing evidence has indicated that testosterone level was significantly lower in men with type 2 diabetes mellitus (T2DM) (1). Reduced testosterone was recognized as an accompanying phenomena of reduced insulin sensitivity (2), poor glycemic control (3), and obesity (4). In our previous study, a short-term intensive insulin therapy of 3-5 days upregulated serum testosterone levels significantly (5), while the underlying mechanisms remain unclear.

In testes, 3β-hydroxysteroid dehydrogenase (3β-HSD) and 17β-hydroxysteroid dehydrogenase (17β-HSD) are the key enzymes in changing the dehydroepiandrosterone (DHEA) into testosterone (6). It has been reported that insulin administration increased testosterone synthesis by stimulating testicular β-HSD activity in streptozotocin-induced diabetic rats (7). *In vitro* studies, insulin stimulated the HSD activity of human placental cytotrophoblasts (8) and ovarian thecal-like tumor cells (9). Therefore, we hypothesized that short-term insulin therapy might increase transformation from DHEA to testosterone *via* regulating β-HSD activity. However, the relationship between insulin therapy and DHEA and β-HSD levels in patients with diabetes has not been reported.

Therefore, we further analyzed the changes of dehydroepiandrosterone sulfate [DHEA-S, the major circulating form of DHEA (10)] and testosterone levels, as well as the 3 and 17 β-HSD activity after intensive insulin therapy in newly diagnosed males with T2DM.

## Materials and methods

### Study design and participants

The present study is a secondary analysis of our previous study (clinicaltrials.gov, NCT03982238) (5). This study recruited men with newly diagnosed T2DM from June 2019 to November 2019. The inclusion criteria were as follows: (1) aged 18-60 years, met the WHO 1999 diagnostic criteria of diabetes, and had not been treated with any hypoglycemic drugs; (2) who had an HbA1c 9.0% (75 mmol/mol) or higher which was recommended to take intense insulin therapy according to the Chinese Guidelines for the prevention and management of T2DM (2017 edition); (3) willingness to follow dietary and exercise advice. Exclusion criteria were as follows: (1) already on treatment with lipid-lowering or anti-hypertensive medications; (2) with serum alanine aminotransferase (ALT) levels more than 2.5 times the upper normal range (100 U/L) or creatinine levels more than 1.3 upper normal range (105 μmol/L); (3) a history of systemic corticosteroids use in the past 3 months; (4) any infection or acute diabetic complication such as ketoacidosis or hyperosmolar state (coma); (5) patients who developed insulin allergy.

### Procedures

After admission, baseline parameters of height, weight, age, and medical history were collected. Body mass index (BMI) was calculated as weight divided by the square of height (kg/m^2^). Blood samples were collected after an overnight fast for assessment of blood glucose, beta-cell function (C-peptide and insulin levels), HbA1c, testosterone, DHEA-S and 3/17 β-HSD. The patients were then started on intensive treatment with continuous subcutaneous insulin infusion (CSII). The patients were tested for capillary blood glucose at least 7 times a day and insulin doses were titrated daily according to blood glucose levels by specialist physicians. In our hospital, this almost always restores the glucose values to the normal range within 3 to 5 days, and no more than a week.

After achieving the required standard of satisfactory blood glucose levels (>80% of values within the range of pre-meal blood glucose 3.9~7.0 mmol/L, postprandial blood glucose 3.9~10.0 mmol/L), measurements of fasting blood glucose, beta-cell function, testosterone, DHEA-S and 3/17 β-HSD were performed again.

### Laboratory tests

Fasting blood glucose was measured by an auto-analyzer (Modular E170; Roche, Mannheim, Germany); HbA1c was measured using high-performance liquid chromatography assay (Bio-Rad Laboratories, USA); C-peptide, insulin, and sexual hormones including total testosterone, DHEA-S, estradiol, luteinizing hormone (LH), follicle-stimulating hormone (FSH), and sex hormone binding globulin (SHBG) were measured using chemiluminescent microparticle immunoassay (Architect system, USA). 3β-HSD and 17β-HSD were measured by enzyme-linked immunosorbent assay (Jiangsu Meimian Industrial, China). Both the intra- and inter-assay coefficients of variation were less than 10%.

### Statistical analysis

All statistical analyses were performed using SPSS version 22.0 software (IBM Corp., USA). All variables were tested for normal distribution. Data are presented as mean (95%CI) or percentage. Changes from baseline to the endpoint of the study were assessed by a paired t-test for parametric data or a Wilcoxon for nonparametric data, respectively. Spearman analysis was used to find the factors which may be correlated with the testosterone/DHEA-S transform. The categorical data were examined with chi-square test. A p value < 0.05 was considered statistically significant.

We tested the 3/17 β-HSD before and after intensive insulin therapy in six subjects in a pre-study. The 3β-HSD increased from 11.8 to 15.0 pg/ml, and the standard deviation was 3.2 pg/ml. The 17β-HSD increased from 15.0 to 26.7 pg/ml, and the standard deviation was 11.7 pg/ml. Therefore, we need at least 13 subjects with 80% power and an α of 0.05. The sample size was calculated using PASS software.

## Results

### 1. Clinical and biochemical characteristics at baseline

A total of 43 males who had data of testosterone and DHEA-S levels were enrolled in the study. The mean age of these patients was 45.7 (42.6, 48.9) years, BMI was 25.5 (24.5, 26.6) kg/m^2^, and the HbA1c was 10.8 (10.3, 11.2) %.

### 2. Changes of serum testosterone and DHEA-S levels

**Figure 1:**
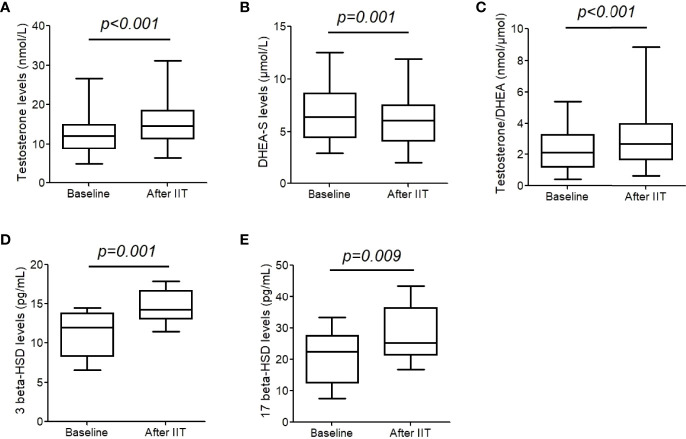
Testosterone, dehydroepiandrosterone sulfate (DHEA-S), and 3/17 β-hydroxysteroid dehydrogenase (HSD) levels before and after intensive insulin therapy (IIT). **(A-C)** testosterone and DHEA-S levels were measure in 43 patients with T2DM. **(D, E)** 3/17 β- HSD levels were measured in 17 patients. The differences between two groups were analyzed were assessed by a paired t-test. The whiskers of the box were showed as 5–95 percentile.

We measured testosterone and DHEA-S levels before and after to explore the change of 3/17 β-HSD activity. After the intensive insulin therapy, testosterone level increased significantly (from 13.0 (11.3, 14.6) nmol/L to 15.7 (13.9, 17.5) nmol/L, *p*<0.001, **Figure 1A**), while DHEA-S level decreased (from 6.5 (5.7, 7.3) μmol/L to 6.0 (5.3, 6.7) μmol/L, *p*= 0.001, **Figure 1B**). Therefore, the ratio of testosterone/DHEA-S increased significantly (2.4 (2.0, 2.8) nmol/μmol vs. 3.1 (2.6, 3.7) nmol/μmol, *p*<0.001, **Figure 1C**). Free testosterone (FT), bioavailable testosterone (Bio-T) were also calculated as reported previously (11). FT increased from 0.21 (0.18, 0.24) nmol/L to 0.26 (0.22, 0.29) nmol/L (*p*<0.001), and Bio-T increased from 4.9 (4.3, 5.4) nmol/L to 5.6 (5.0, 6.1) nmol/L (*p* = 0.001).

### 3. Changes of serum 3/17β-HSD concentrations

We further performed serum 3 and 17 β-HSD concentrations in 17 males. After blood glucose normalization with the short-term CSII therapy, 3β-HSD increased from 11.0 (9.5, 12.5) pg/mL to 14.6 (13.5, 15.7) pg/mL, *p*= 0.001, and 17β-HSD increased from 20.7 (16.3, 25.2) pg/mL to 28.2 (23.8, 32.5) pg/mL, *p*= 0.009 (**Figures 1D, E**).

### 4. Changes of other sexual hormones

**Figure 2:**
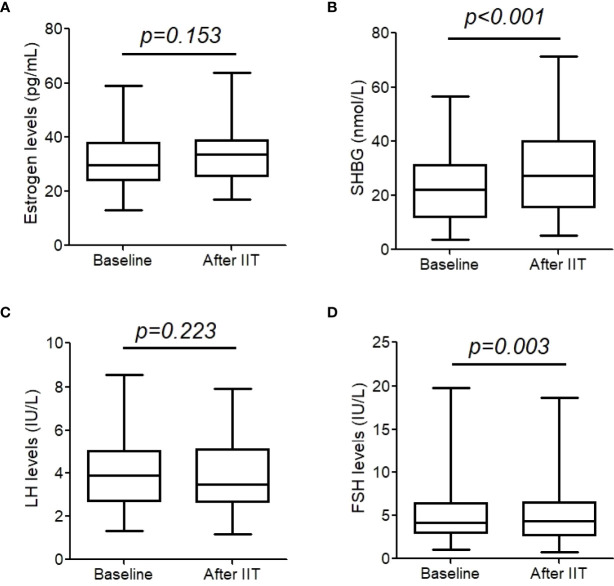
Estrogen **(A)**, sex hormone binding protein (SHBG) **(B)**, luteinizing hormone (LH) **(C)**, and follicle-stimulating hormone (FSH) **(D)** before and after intensive insulin therapy (IIT). These hormones were measure in 43 patients with T2DM. The whiskers of the box were showed as 5–95 percentile.

As shown in **Figure 2**, estrogen and LH did not change significantly after intensive insulin therapy (both *p >*0.05). However, SHBG significantly increased (22.9 (19.2, 26.7) nmol/L vs. 28.8 (24.5, 33.0) nmol/L, *p*< 0.001), and FSH decreased from 5.3 (4.2, 6.4) IU/L to 5.0 (4.0,6.1) IU/L, *p* = 0.003.

### 5. Changes of blood glucose and insulin levels

The average insulin dose used in the insulin pump was 0.6 (0.5,0.7) IU/kg per day when the blood glucose normalized. Fasting blood glucose decreased from 9.6 (8.7, 10.5) mmol/L to 6.1 (5.5, 6.6) mmol/L, *p*<0.001. Fasting insulin levels increased from 9.5 (7.8, 11.1) IU/L to 15.0 (12.1, 18.0) IU/L (*p*< 0.001), and C-peptide decreased from 1.5 (1.2, 1.7) ng/mL to 0.6 (0.5, 0.7) ng/mL (*p*= 0.007).

### 6. Factors that may influence the ratio of testosterone/DHEA-S

Spearman analysis showed that the change of testosterone/DHEA-S (the ratio of testosterone/DHEA-S after intensive insulin therapy minus that at baseline, Δtestosterone/DHEA-S) was correlated with Δ17β-HSD (r = 0.571, *p* = 0.013). The correlation between Δtestosterone/DHEA-S and Δ3β-HSD was not statistically significant (r = 0.401, *p* = 0.099), and there were no significant differences between Δtestosterone/DHEA-S and insulin dose at the time point of blood glucose normalization (r = -0.140, *p* = 0.578), the change of serum insulin levels (r = -0.051, p = 0.830), and the change of fasting blood glucose (r = 0.006, *p* = 0.972).

## Discussion

The present study demonstrates for the first time that in men with newly diagnosed T2DM, short-term insulin therapy upregulated testosterone level, accompanied by a decreased DHEA-S level. Insulin administration enhanced the activities of 3β-HSD and 17β-HSD, which may at least partly explain the mechanisms of insulin therapy on testosterone elevation.

A number of studies have confirmed that poor glycemic control and insulin resistance lead to low testosterone levels, testosterone deficiency, and male hypogonadism (12, 13). Intensive insulin therapy can improve glycemic control effectively and rapidly in patients with T2DM (14). In the present study, we found that in male patients with newly diagnosed T2DM, intensive insulin therapy within a week increased their circulating testosterone levels, including total testosterone, FT and Bio-T levels. The results confirmed our previous study (5). Gagliano et al. found that in non-diabetic subjects, ingestion of either a glucose load or a mixed meal resulted in a significantly increased blood glucose as well as a decrease in serum total testosterone levels, suggesting that changes in blood glucose can have a rapid and significant effect on testosterone levels (15). While in males with obesity and T2DM, researchers believed that weight loss more than glycemic control may improve testosterone levels (16). They found that circulating free testosterone increased after bariatric surgery (17) as well as exenatide therapy (18, 19). However, in our study, CSII therapy for a week increased serum testosterone without significant weight change, suggesting that, in those subjects, the elevated testosterone levels are mainly due to the improved glycemic control or direct action of insulin on Leydig cells.

DHEA, a steroid hormone produced mainly by the adrenal cortex, works as the most important precursor for testosterone production. Therefore, DHEA has been aggressively sold as a dietary supplement to boost testosterone levels (20). In Leydig cells, DHEA-S is hydrolyzed back to DHEA by steroid sulfatase for further conversion. Firstly, DHEA is oxidized by 3β-HSD on the 3β-hydroxy group to generate androstenedione, later then catalyzed by 17β-HSD and results in a reduction of its C17 keto group to a β-hydroxyl group, and finally generates testosterone (10). Interestingly, it has been reported that insulin reduces serum DHEA and DHEA-S in men either by inhibiting their production or by increasing the metabolic clearance rate of DHEA (21). In addition, patients with T2DM, whose serum insulin levels were high, had significantly lower DHEA and DHEA-S serum levels than normal subjects and patients with T2DM whose serum insulin levels were normal (22). In PCOS rat models induced by DHEA administration, serum testosterone concentrations significantly increased with DHEA administration, but the increase was inhibited by oral administration of insulin-lowering agents (23). In the present study, we also found a reduction of serum DHEA-S levels in those newly diagnosed males with T2DM after insulin administration, which means that DHEA has a contradictory tendency to testosterone under insulin therapy, indicating that insulin may promote the conversion from DHEA to testosterone.

Previous studies showed that STZ induced diabetic rats have a marked reduction in serum testosterone as well as the activities of Leydig cellular 3β-and 17β-HSD, while insulin treatment reversed these changes (24). Our present study directly confirmed the activation of 3β-and 17β-HSD by short-term insulin therapy in humans *in vivo* for the first time. Certain commercially available drugs, such as gentamicin, can decrease the basal plasma testosterone concentration through inhibiting 3 and 17β HSD activity (25), while Ginkgo biloba extract can induce an ascending tendency of the expression of 17β-HSD3 and 3β-HSD1 and significantly increased the concentrations of serum testosterone levels in patients with T2DM (26). Thus, it is quite possible that the upregulating effect of insulin on serum testosterone levels as well as the downregulating effect on DHEA, are mainly dependent on the enhanced activities of 3β-HSD and 17β-HSD, especially 17β-HSD according to our correlation analysis. In future, further research is needed to understand the effect of insulin on testosterone.

The effects of DHEA and DHEA-S other than sex hormones have attracted increasing attentions recently. A comment in *Nature* reported a study that the combination of growth hormone, metformin, and DHEA treatment can reverse biological age (27, 28). In male patients with T2DM, low serum DHEA-S concentration has reported as a predictor for deterioration of urinary albumin excretion (29). Previous study also showed that serum DHEA-S concentration was negatively associated with carotid atherosclerosis in men with type 2 diabetes (30). DHEA and DHEA-S present protective actions on the cardiovascular system (31). Moreover, DHEA may have acute effects to protect against hypoglycemia-associated neuroendocrine and autonomic failure in healthy humans (32). Thus, for patients who are receiving insulin therapy, whether DHEA replacement, rather than testosterone may have long-term benefit deserves further prospective study.

Consistent with previous studies, insulin therapy increased serum SHBG levels (33). Studies *in vivo* have shown that metabolic disturbances can decrease SHBG production through inhibiting HepG2 expression (34). Therefore, the upregulated SHBG level after insulin therapy may be due to the improvement of glycemic control. In the present study, FSH were decreased after insulin therapy. FSH mainly stimulates Sertoli cells to secrete androgen-binding protein (35), and also increases testosterone levels by amplifying the LH response through the 17β HSD (36). Therefore, we speculated that the elevation of 17β HSD and SHBG may inhibit FSH levels through a feedback regulatory mechanism. However, the reason of FSH reduction after insulin therapy needs further study. It is a pity that we failed to find any correlation between the blood glucose or insulin dose with the change of testosterone/DHEA, the possible reason may be due to the limited sample size. In addition, another group of subjects, which achieves the standard glycemic range through dietary control only, should be set up to further clarify the regulation mechanism of insulin on testosterone, either due to the direct effect of insulin itself or secondary to the decreased glycemic level.

In conclusion, short-term insulin intensive therapy in patients with T2DM can increase 3 and 17 β-HSD levels, which leads to elevation of testosterone and reduction of DHEA levels. Given the importance of DHEA for health, further studies are necessary to verify the present results by replicating them in a longer-term randomized controlled study.

## Data availability statement

The original contributions presented in the study are included in the article/supplementary material. Further inquiries can be directed to the corresponding authors.

## Ethics statement

The studies involving human participants were reviewed and approved by Institutional Ethics Committee of Nanjing First Hospital, Nanjing Medical University. The patients/participants provided their written informed consent to participate in this study.

## Author contribution

Conceptualization: BD, J-HM. Data curation: YH, YW, T-TC, LL, D-ML. Formal analysis: YW, YH. Funding acquisition: J-HM. Investigation: D-ML. Methodology: YH, BD. Project administration: J-HM, BD. Resources: J-HM. Software: YH. Supervision: J-HM. Validation: J-HM. Visualization: YH. Writing – original draft: YW. Writing – review and editing: YH. All authors contributed to the article and approved the submitted version.

## Funding

This study was supported by the National Natural Science Foundation of China (No. 81870563).

## Conflict of interest

The authors declare that the research was conducted in the absence of any commercial or financial relationships that could be construed as a potential conflict of interest.

## Publisher’s note

All claims expressed in this article are solely those of the authors and do not necessarily represent those of their affiliated organizations, or those of the publisher, the editors and the reviewers. Any product that may be evaluated in this article, or claim that may be made by its manufacturer, is not guaranteed or endorsed by the publisher.
